# The Association between Maltreatment in Childhood and Pre-Pregnancy Obesity in Women Attending an Antenatal Clinic in Australia

**DOI:** 10.1371/journal.pone.0051868

**Published:** 2012-12-31

**Authors:** Katharine Hollingsworth, Leonie Callaway, Michael Duhig, Sally Matheson, James Scott

**Affiliations:** 1 Metro North Mental Health, Royal Brisbane and Women's Hospital Herston, Herston, Australia; 2 Department of Medicine, Royal Brisbane and Women's Hospital Herston, Herston, Australia; 3 School of Medicine, The University of Queensland, Herston, Australia; 4 Queensland Centre for Mental Health Research, The Park Centre for Mental Health, Wacol, Australia; 5 The University of Queensland Centre for Clinical Research, Herston, Australia; University of Missouri-Kansas City, United States of America

## Abstract

**Introduction:**

Obesity in pregnancy is associated with increased risk of complications and adverse outcomes in mother and child. Childhood adverse experiences are known to have numerous negative physical and emotional sequelae. We aimed to examine if exposure to abuse and/or neglect in childhood increased the likelihood of pre-pregnancy obesity.

**Methods:**

Demographic and clinical data including weight, height, mental health as measured by the General Health Questionnaire and exposure to childhood trauma as measured by the childhood trauma questionnaire was collected from 239 women attending antenatal care at an Australian tertiary hospital.

**Results:**

More than one quarter of women were obese prior to pregnancy and approximately 20% of women self reported experiencing moderate to severe physical, sexual or emotional abuse. Almost 60% of women scored in the clinical range on the GHQ. Pre-pregnancy obesity in women attending antenatal care was associated with a self-reported history of emotional or physical abuse with those exposed to moderate or severe emotional or physical abuse having increased odds of being obese prior to pregnancy (O.R. and 95% CI: 2.40; 1.19–4.84 and 2.38; 1.18–4.79 respectively). There was no significant association between other forms of childhood maltreatment, demographic or current mental health status and pre-pregnancy obesity.

**Conclusions:**

The high rates of obesity, mental health problems and self reported childhood maltreatment in the Australian antenatal population are serious public health concerns due to the extra health risks conferred on mother and offspring. Exposure to physical or emotional abuse during childhood increases the likelihood of obesity in women attending antenatal care. Further research is required to determine reasons for this association.

## Introduction

The prevalence of obesity is increasing worldwide [1] with associated adverse impacts across a range of health outcomes. Consistent with this trend, there has been an increase in rates of obesity in pregnant women over the past two decades [2]–[4]. Women of childbearing age (18–36 years) have a higher rate of weight gain than those in other age groups [5]. Maternal obesity has significant negative effects on fertility, the developing foetus, maternal health during pregnancy and delivery as well as ongoing negative health implications for the child through to adulthood [6]–[9].

Exposure to childhood maltreatment (abuse or neglect) has numerous, well-documented negative sequelae with increased risk of a wide range of later mental and physical health problems [10]–[14] including obesity [15]. For example, Lissau and Sorensen (1994) found that parental neglect in childhood was a significant risk factor for adult obesity independent of age, social background and increased body mass index (BMI) in childhood [16]. Pederson and Wilson (2009) found a correlation between childhood neglect and adult obesity in women. In this study, depression was a mediating factor in the association. In addition to neglect, a positive association between childhood sexual abuse and adult obesity has been reported [10], [11].

These studies have generally examined one exposure to adversity (e.g. neglect) with obesity. Childhood trauma, however, is interrelated [17]. It is uncommon for people with a history of childhood trauma to be exposed to a single type of adversity. Studies examining the association between adult obesity and childhood trauma are therefore required, to explore different types of childhood adversity [18]. In one of the few studies exploring the correlation between childhood trauma and obesity that did not rely on retrospective reporting of childhood abuse and neglect, Johnson and Cohen (2002) found that a wide range of childhood adversities were associated with adolescent and early adulthood problems with obesity [19].

Our a priori hypothesis was that exposure to any form of childhood abuse or neglect would increase the risk of pre-pregnancy obesity. This study aimed to explore if self reported exposure to maltreatment in childhood was associated with pre-pregnancy obesity in a sample of mothers attending antenatal care.

## Methods

Women attending their antenatal appointments at the Royal Brisbane and Women's Hospital were invited to participate in the research. Participants were recruited from all outpatient antenatal clinics- including general obstetric and midwife clinics, endocrine clinics, high-risk pregnancy clinics, obstetric medicine clinics, and drug and alcohol obstetric clinics. Recruitment was conducted over two separate periods in 2008 and 2009. Consecutive, unselected women were individually approached by the researcher (KH) while they were waiting for their appointment. Consenting participants were included if they were currently pregnant, of any gravidity or gestational stage, and were excluded if they were under 18 or unable to provide informed consent to the study because of reading difficulties or intellectual disability. Participants were advised verbally and in writing of the purpose of the study and the contents of the questionnaire prior to obtaining consent. Ethics approval for this study was obtained from the Royal Brisbane and Women's Hospital Human Research Ethics Committee. While we attempted to include all women attending the clinic, we were not able to accurately measure all those who declined to participate in a large and busy outpatient clinic.

Women who consented to participation completed self-report questions, which collected demographic data, information regarding weight, height, their current and previous pregnancies, the General Health Questionnaire (GHQ) and the Childhood Trauma Questionnaire (CTQ). The GHQ is a well-validated 12 item self reported screening instrument used to detect mental health problems in the community and also in non-psychiatric clinical settings such as primary care or general practice. It assesses an individual's mental health functioning across a number of areas including anxiety, depression and social dysfunction within past three weeks. An example of a GHQ item is “Have you recently felt constantly under strain” [20]. Responses are scored on a four-point Likert- type scale, giving possible scores between 0–36. A score of 12 or greater indicates that the person is in the clinical range for mental health problems [21]. The CTQ is a widely used 28 item self report instrument with questions that ask of exposure to abuse and neglect in childhood [22], [23]. It was chosen as it examines emotional, physical and sexual abuse and emotional and physical neglect. An example of a CTQ item is “*People in my family called me things like “stupid”, “lazy”, or “ugly””*. This is scored on a 5 point Likert scale, with options ranging from *“rarely true”* ( = 1) to *“very often true”* ( = 5), producing scores from 5 to 25 for each trauma subscale. The CTQ was scored according to the manual, which classifies responses to each category of abuse as “none to minimal”, “low to moderate” and “moderate to severe”, depending on the participant's responses to items in the trauma subscale [22].

### Statistical Analysis

Subjects were categorised into obese (Pre-pregnancy BMI >30) and non-obese. The association between pre-pregnancy obesity and continuous variables (age, GHQ and CTQ scores) was analysed using independent sample t-tests. The associations between pre-pregnancy obesity and categorical variables (completion of high school, household income, relationship and employment status, exercise and parity) were analysed using a Chi Square goodness of fit. Household income was divided by the median split into less than or equal or greater than $75 000/annum. Parity was categorised into those women who were nulliparous compared to those who had a previous delivery and relationship status was grouped into those who reported they were in a relationship compared to those who were not.

Self reported response to each type of abuse or neglect was then categorised into “none or minimal”, “low to moderate” and “moderate to severe” in accordance with the CTQ manual [22]. The association between pre-pregnancy obesity and different types of trauma was examined using logistic regression. We used this analysis in order to quantify the odds of pre-pregnancy obesity when exposed to each type of childhood trauma.

Post hoc sensitivity analyses were undertaken. Initially, we explored if any form of maltreatment was associated with pre-pregnancy obesity. Two levels of maltreatment were established from the CTQ; absent (previously defined as non to minimal) or present (previously defined as low to moderate or moderate to severe). The association between any childhood maltreatment and pre-pregnancy obesity was then analysed. Moreover, as children who are exposed to childhood maltreatment are at increased risk of eating disorders [24], we excluded women who were classified as underweight or severely underweight (BMI ≤19.9) and repeated analyses. Finally, we examined if an association was identified between childhood maltreatment and being overweight pre-pregnancy (BMI ≥25.00–29.99) *or* being obese pre-pregnancy (BMI ≥30).

## Results

A total of 239 women participated in the study. The average age was 29.2 years (*SD*  = 5.5 years). Most participants were either married (54%) or in a defacto relationship (33.9%). One third of subjects were nulliparous and almost half had either one or two previous deliveries. The mean pre-pregnancy weight was 71.9 kgs (*SD*  = 18.3 kgs, *Range  = *40 kgs – 150 kgs). The mean pre-pregnancy BMI was 26.24 (*SD*  = 6.32, *Range*  = 16.02 and 60.09) and the median was 26.82. The percentage of participants in each of the BMI categories was; severely underweight (BMI =  <18.5), 3.3% (n = 8); underweight (BMI  = 18.6–19.9), 7.9% (n = 19); healthy weight (BMI = 20.0–24.9) 41% (n = 98); overweight (BMI = 25.0–29.9), 21.3% (n = 51) and obese (BMI = 30.0>), 26.4% (n = 63) [22]. Using a GHQ clinical threshold of 11 [22] 140 (58.6%) participants scored in the clinical range. There was a trend for those participants with pre-pregnancy obesity to have not completed high school (χ^2^ = .58, *p* = .058). Otherwise, there were no significant differences in the demographic variables or GHQ scores in the women with pre-pregnancy obesity and those who were not obese. Similarly, the total scores on the childhood trauma questionnaire were not significantly different between these two groups of women as illustrated in table 1.

**Table 1:** Comparison of demographic and clinical factors in women who were obese compared to those who were not obese before becoming pregnant.

	Obese (N = 63)	Non-Obese (N = 176)	Statistic
Age	28.8 (SD = 5.24)	29.4 (SD = 5.63)	*t* ^235^ = .67, *p* = .504
GHQ score	12.44 ( SD = 6.42)	12.81 (SD = 5.99)	*t* ^237^ = .40, *p* = .686
Completed high school	66.7% (42/63)	79.5% (140/176)	χ^2^ = .58, *p* = .058
Currently Employed	50.8% (32/63)	54% (95/176)	χ^2^ = .66, *p* = .664
In a relationship	88.9% (56/63)	87.5% (154/176)	χ^2^ = .77, *p* = .772
$75,000+ Household income	54.2%(32/59)	54.8% (86/157)	χ^2^ = .97, *p* = .977
Nulliparous	34.4% (21/61)	37.2% (58/156)	χ^2^ = .70, *p* = .696
Exercise 2 or more times a week	65% (39/60)	71.8% (125/174)	χ^2^ = .32, *p* = .318
Total Abuse	45.32 (SD = 32.17)	40.98 (SD = 39.07)	*t* ^237^ * = *−.790, *p* = .431

In exploring different types of childhood maltreatment, emotional abuse was a common experience in the sample self reported by 88 (36.9%) participants. Emotional abuse was associated with pre pregnancy obesity in a dose response fashion. Those reporting moderate to severe emotional abuse were almost two and a half times more likely to be obese before pregnancy (OR and 95% CI: 2.40; 1.19–4.84) compared to women who reported experiencing no or minimal emotional abuse. Moderate to severe physical abuse was experienced by 43 (18.0%) of the 238 women who completed the questionnaire. These subjects were also more likely to be obese compared to women who reported none or minimal physical abuse (OR and 95% CI: 2.38; 1.18, 4.79). Some form of sexual abuse was reported by almost one quarter of the sample (58 of 237 subjects). There was no significant association between sexual abuse and pre-pregnancy obesity. Similarly there was no significant association between emotional or physical neglect and pre-pregnancy obesity. The relationship between pre-pregnancy obesity and childhood abuse and neglect is shown in Table 2.

**Table 2:** Association between obesity and childhood abuse and neglect.

Variable	Non-Obese N (%)	Obese Proportion N (%)	OR
*Emotional Abuse*			
None to minimal	150 (63.0)	34 (22.7%)	1.00
Low to moderate	42 (17.7)	10 (23.8%)	1.07 (0.48, 2.34)
Moderate to severe	46 (19.3)	19 (41.3%)	2.40* (1.19, 4.84)
*Physical Abuse*			
None to minimal	172 (72.3)	40 (23.3%)	
Low to moderate	23 (9.7)	5 (21.7%)	0.92 (0.32, 2.63)
Moderate to severe	43 (18.1)	18 (41.9%)	2.38* (1.18, 4.79)
*Sexual Abuse*			
None to minimal	179 (75.2)	42 (23.5%)	
Low to moderate	11 (4.6)	5 (45.5%)	2.72 (0.79, 9.36)
Moderate to severe	47 (19.7)	25 (31.9%)	1.53 (0.76, 3.09)
*Emotional Neglect*			
None to minimal	158 (66.4)	37 (23.4%)	
Low to moderate	51 (21.4)	19 (37.3%)	1.94 (0.99, 3.82)
Moderate to severe	28 (11.8)	6 (21.4%)	0.89 (0.37, 2.36)
*Physical Neglect*			
None to minimal	192 (80.7)	47 (24.5%)	
Low to moderate	19 (8.0)	7 (36.8%)	1.80 (0.67, 4.84)
Moderate to severe	19 (8.0)	7 (26.3%)	1.37 (0.56, 3.36)

* =  *p*<.05.

The initial sensitivity analysis concluded that when childhood maltreatment was dichotomised into present or absent, no significant associations between childhood maltreatment and pre-pregnancy obesity were identified. When women with a pre-pregnancy BMI of ≤19.9 were excluded from analysis, the association between moderate to severe emotional abuse and pre-pregnancy obesity remained (OR and 95% C.I. 2.66; 1.22, 5.76), however, the association between moderate to severe physical abuse and pre-pregnancy obesity was no longer significant (OR and 95% C.I. 2.00; 0.94, 4.26). Finally, when overweight and obese pre-pregnancy BMI classified women were compared to those of a healthy pre-pregnancy weight (BMI ≥20–24.99) all associations between childhood maltreatment and pre-pregnancy weight lost significance.

## Discussion

It was common for women attending antenatal care to be overweight or obese prior to their pregnancy. Obesity in this population is associated with elevated risks of adverse health outcomes to both the mother and the unborn offspring increasing the likelihood of medical interventions during pregnancy and delivery [6]–[9]. For these reasons, prevention or intervention to reduce obesity in women of child bearing age are especially important.

Mental health problems were another concern in this sample. Almost 60 percent of mothers scored greater than 11 on the General Health Questionnaire placing them in the clinical range for mental health problems [21]. In an Australian community sample of females of child bearing age, 26.4% scored in the clinical range of the GHQ [25], [26]. However, in an Australian study examining the effect of exercise on psychological well being of 72 women attending either hospital or community pre-natal classes, 44% of women scored in the clinical range of the GHQ [27]. In a much larger study of Australian pregnant (<24 weeks gestation) nulliparous women, 68% had three or more health complaints, the most common being exhaustion, nausea, back pain, constipation and severe headaches [28]. Consistent with our current study, women have high levels of symptoms affecting both mental and physical health. Mental health problems during pregnancy may have serious adverse effects to both mother and offspring [29]–[31].

Finally, childhood abuse and neglect were common in this sample of women attending antenatal care. The most commonly self reported form of maltreatment during childhood was emotional abuse experienced by almost 50% of the sample. Although physical neglect was the least common form of maltreatment reported by participants, it was still reported by one in six participants. These rates are high compared to other studies of childhood trauma in Australian populations [32]–[35]. The high rates of trauma may be due to the sample studied. The women were attending a public tertiary hospital. Sampling occurred from all antenatal clinics including specialist antenatal clinics such as drug and alcohol obstetric clinics where women may have experienced substantially higher rates of adversity in childhood.

This study found that pre-pregnancy obesity in mothers attending an antenatal clinic was associated with a self-reported history of emotional or physical abuse. There was a dose dependent response with those exposed to moderate or severe emotional or physical abuse having increased odds of being obese prior to pregnancy. Sensitivity analyses revealed that there was no association identified between childhood maltreatment and pre-pregnancy obesity when childhood maltreatment was dichotomised into present or absent. This suggests that only the exposure to the more severe physical or emotional abuse was associated with pre-pregnancy obesity. When overweight and obese pre-pregnancy categories were included as the outcome variable, the previous association with moderate or severe emotional and physical abuse lost significance. This suggests that the moderate to severe forms of physical and emotional childhood maltreatment were associated with pre-pregnancy obesity (BMI≥30) but not with being overweight pre-pregnancy (BMI ≥20–24.99).

There have been a number of mechanisms postulated for the link between childhood trauma and obesity [36]. There is significant evidence showing that childhood abuse and trauma is related to disordered eating (including binge eating) and impaired impulse control in teenagers and adults [11], [36]. Childhood trauma is known to lead to higher body dissatisfaction which is associated with greater weight variation and poorer outcomes in weight management programs [37], [38]. Eating in response to negative emotions and stress predicts poor outcome in terms of weight control [39]. There is considerable evidence to indicate that early life trauma leads to overactivation of the hypothalamic-pituitary axis, resulting in excess adipose deposition [12], [36], [40]. The most likely explanation for the lack of a significant association in this study between sexual abuse and pre-pregnancy obesity was the relatively small sample rise. Other studies have reported an association between sexual abuse in childhood and adult obesity [10], [11]. We believe our study was underpowered to show a significant association resulting in a type II error. Further research is needed to clarify the mechanisms underpinning the association between adversity in childhood and adult obesity.

The findings of this study have a number of clinical implications. Firstly, a high proportion of women attending the antenatal clinic were overweight or obese. These rates of pre-pregnancy obesity are higher than those reported in a study of women attending an antenatal clinic of a tertiary teaching hospital between 1998 and 2002 where only 34% of women were overweight or obese [4]. The rates of obesity in this study are consistent with Australian Bureau of Statistics data from 2007–2008 which reported 44.37% of women aged 25–34 were overweight or obese [41]. Just as mothers are now better informed of the risks of smoking and alcohol use during pregnancy [42], [43], education campaigns may assist in informing women of the increased risks associated with obesity to mother and offspring. This needs to be accompanied by affordable and accessible programmes to assist with weight reduction in women planning to have children [44]. Secondly, rates of exposure to childhood trauma remain at unacceptably high levels and the association with later obesity shows the negative health effects continue into adulthood and may well affect the physical health of the next generation. There is an urgent need for evidence based interventions to be implemented to reduce the risk of maltreatment to Australian children [45]–[49]. Finally, childhood trauma has a tendency to be transgenerational. The children of parents who have experienced childhood trauma are at increased risk of exposure to adversity in childhood [18]. Thus the offspring of mothers who have been exposed to childhood maltreatment are at high risk of exposure to childhood trauma. There is an argument for offering non-stigmatising support and interventions for these ‘at risk’ families [49], [50].

One of the major problems with much of the research into the adult sequelae of childhood trauma and neglect, including this study, is that the majority of these studies are retrospective and rely on recall of childhood trauma [50]. It has been argued that it is likely that traumatic events in childhood are under-reported [51]. A further limitation of this study is that it is a cross sectional assessment of associations based on retrospective information. No information is available regarding the timing or duration of abuse. There is also no available information regarding other factors in childhood, including childhood weight. It is therefore not possible to conclude in our study that the maltreatment preceded obesity nor to independently verify information pertaining to maltreatment provided in the anonymous self-report questionnaires. Pre-pregnancy BMI was a self-reported estimate and may be inaccurately recalled by women at various stages of their pregnancies [52], [53]. The response rate and the characteristics of non-responders are not known. Women were informed of the aims of the survey possibly leading to a bias of those who consented to participate [51]. Finally, our sample size varied with some participants not answering all items on the survey. However, these numbers were small and unlikely to have affected the findings.

This study found that mental health problems, exposure to childhood maltreatment and pre-pregnancy obesity were all common in this sample of Australian women. Education and interventions are needed to address these serious public health concerns. Further research is needed in order to explain the reasons for the association between physical or emotional abuse and obesity.
